# Towards Optical Partial Discharge Detection with Micro Silicon Photomultipliers

**DOI:** 10.3390/s17112595

**Published:** 2017-11-10

**Authors:** Ming Ren, Jierui Zhou, Bo Song, Chongxing Zhang, Ming Dong, Ricardo Albarracín

**Affiliations:** 1 State Key Laboratory of Electrical Insulation for Power Equipment, Xi’an Jiaotong University, 28 Xianning West Road, Xi’an 710049, China; ronaldo@stu.xjtu.edu.cn (J.Z.); songbo111@stu.xjtu.edu.cn (B.S.); zhangcx.123@stu.xjtu.edu.cn (C.Z.); dongming@xjtu.edu.cn (M.D.); 2 Departamento de Ingeniería Eléctrica, Electrónica, Automática y Física Aplicada, Escuela Técnica Superior de Ingeniería y Diseño Industrial (ETSIDI), Universidad Politécnica de Madrid (UPM), Ronda de Valencia 3, 28012 Madrid, Spain

**Keywords:** optical detection, silicon photomultiplier, partial discharge, gas insulation, PD monitoring

## Abstract

Optical detection is reliable in intrinsically characterizing partial discharges (PDs). Because of the great volume and high-level power supply of the optical devices that can satisfy the requirements in photosensitivity, optical PD detection can merely be used in laboratory studies. To promote the practical application of the optical approach in an actual power apparatus, a silicon photomultiplier (SiPM)-based PD sensor is introduced in this paper, and its basic properties, which include the sensitivity, pulse resolution, correlation with PD severity, and electromagnetic (EM) interference immunity, are experimentally evaluated. The stochastic phase-resolved PD pattern (PRPD) for three typical insulation defects are obtained by SiPM PD detector and are compared with those obtained using a high-frequency current transformer (HFCT) and a vacuum photomultiplier tube (PMT). Because of its good performances in the above aspects and its additional advantages, such as the small size, low power supply, and low cost, SiPM offers great potential in practical optical PD monitoring.

## 1. Introduction

Partial discharges (PDs), which are the potential hazard for insulation, commonly accompany a range of physical phenomena, such as rapid current flow, electromagnetic (EM) radiation, and mechanical vibration [1]. Based on these phenomena, the current induction method [2], ultra-high frequency (UHF) method [3,4], and acoustic emission (AE) method [4,5] were developed for on-site PD monitoring, and many studies were conducted to suppress the EM or the acoustic noise in wide frequency bands to improve the effectiveness of PD detections [6,7,8]. Beyond the aforementioned phenomena, PDs are always accompanied by light radiation during the entire process from primary ionization to extinction. Thus, light radiations are considered the intrinsic nature of PD, because of which, optical detection is a more intuitive means to study the basic properties of discharges, such as discharge morphology, discharge energy, and excitation spectra, and is hardly affected by EM or acoustic noise. In general, the light emission of PD lasts for a notably short-time, which is as low as several picoseconds [9], with low intensity and must be coupled by a system with high-sensitivity, a wide spectral response and a high-time resolution. For a long time, precise optical PD detections are only performed in laboratory studies. For imaging, it generally consists of a spectrometer, a high-shutter-speed charge coupled device (CCD), a microchannel plate (MCP), and a cooling module, and for photon counting, commonly a vacuum photomultiplier tube (PMT) with a high-voltage power supply (several kV) [10]. In the practice of corona detection for outdoor insulation, solar blind ultraviolet (UV) light measurement has been developed based on the physical fact that all solar UV radiation in the range of 240–280 nm is absorbed by the ozone layer, so the entire detected UV radiation originates from the electrical corona.

For PDs in gas insulation systems (e.g., gas-insulated switchgear (GIS), gas-insulated line (GIL), gas-insulated transformer (GIT) and enclosed switch cabinet), the PD light emission is in a dark background without sunlight interference; thus, it is supposed to be effectively detected in a wide spectral range. However, because these laboratory instruments have a large size/weight, high system complexity, and high-cost, a highly sensitive optical PD detection can hardly be realized for actual power equipment. In recent years, the acoustic-optical sensor is developed with microelectromechanical systems (MEMS) technology and is proposed for PD detection [11,12,13]. This kind of sensor is based on the acoustic emission accompanied with PDs, but it is not based on a directly optical approach. The optical-only PD detection has not been fully developed.

With the development of the silicon solid-state photoelectric technology [14], single-photon-level photosensitive devices have been produced with micro-packaging. Solid-state silicon photomultiplier (SiPM) is such a photon-sensitive sensor at millimeter scale in size, which is composed of a dense array of single-photon avalanche diodes (SPAD) [15], each of which operates in Geiger mode. The SiPM sensor is superior in many aspects, such as the high quantum efficiency (up to 50% at low over-voltage of several volts), high responsivity (up to 10^6^ A/W in a wide range of wavelength), broad response spectral range (from ultraviolet to near-infrared), high EM-interference immunity, and a high-level of integration (the microcell density of the SPAD is up to several 1000/mm^2^) [16], which makes the SiPM sensor a highly attractive alternative to the conventional vacuum PMT and other photoelectric devices. The SiPM sensor has rapidly gained proven performances in a wide range of fields, including medical imaging [17], hazard and threat detection [18], high-energy physics and astronomy [19], and light detection and ranging (LiDAR) [20]. We can expect that this photoelectric sensing technology will be gradually applied to electric engineering fields, such as dielectric material characterization, insulation status diagnosis, and power protection system. In particular, the small size of the SiPM sensor enables the built-in optical PD monitoring in gas insulation systems.

To promote the practical application of the optical approach in actual power equipment, a silicon photomultiplier (SiPM)-based PD detection is introduced for the first time to our knowledge in this paper. The principle and optimization of the SiPM based PD detector are introduced in Section 2. In Section 3.1 and Section 3.2, the basic properties of the SiPM-based PD detection, which include the sensitivity, pulse resolution, correlation with PD severity, and EM interference immunity, are experimentally evaluated. Then, in Section 3.3, the quantitative relationship between the SiPM light intensity and the apparent current pulse is analyzed in the entire PD active range. In Section 3.4, the stochastic phase-resolved PD (PRPD) patterns for three typical insulation defects obtained from the SiPM PD detection are compared with those obtained using the high-frequency current transformer (HFCT) and vacuum photomultiplier tube (PMT). The conclusions are drawn in Section 4.

## 2. Experimental Setup

### 2.1. Silicon Photomultiplier Sensor

Silicon photomultiplier (SiPM) integrates a dense array of microcells of avalanche photodiode (APD) and quench resistor, each of which can independently work in a Geiger avalanche mode and fires in response to an absorbed photon. During the photoelectrons flow, a voltage drop across the quench resistor can be coupled. The response time for the microcell to work in the Geiger avalanche mode and the recovery time to recharge to the full operating voltage are essential for PD detection because a PD event lasts for a notably short time (from tens of picoseconds to hundreds of nanoseconds). The Geiger avalanche is confined to the single microcell that is initiated in the avalanche process, during, which all other microcells remain fully charged and ready to detect photons, which ensures that a complete PD photon emission process can be coupled. 

The signal-to-noise ratio (SNR) of the photon detection based on SiPM is impacted by many factors, among which the photon detection efficiency (PDE) and the dark-count-rate (DCR) determine the intrinsic responsivity and the device noise of the sensor, respectively. PDE presents the statistical probability that an incident photon interacts with a microcell to produce an avalanche and it can be calculated by [16]:

(1) $$\mathrm{PDE}=\frac{R\cdot h\cdot c}{\lambda \cdot G\cdot e\cdot P}\cdot 100\%,$$

where *R* is the responsivity of the sensor, which is defined as the average photon-current produced per unit optical power (A/W), and it can be obtained by dividing the measured photo-current by the incident optical power over a particular wavelength; *h* is Planck constant, 6.626 × 10^−34^ J·s; *c* is the speed of light, 3 × 10^8^ m/s, *λ* is the wavelength of the incident light; *e* is the elementary charge, 1.602 × 10^−19^ C, *P* is the probability of over-counting a photon; and, *G* is the primary gain of an SiPM, and defined as the amount of charge created for each detected photon and is a function of the over-voltage and microcell size:

(2) $$G=\frac{C\cdot \Delta U}{e},$$

where *C* is the capacitance of the microcell (F) and Δ*U* is the over-voltage (V) beyond the breakdown voltage (*U_br_*) applied on the cathode of the SiPM. The total applied voltage (*U*_bias_) is calculated by

(3) $$U_{bias}=U_{br}+\Delta U,$$

DCR is the main source of noise in a SiPM and present as current pulses, which is primarily due to thermal electrons generated in the active volume rather than external light. The DCR is a function of temperature as well as the applied over-voltage Δ*U*. 

**Figure 1:**
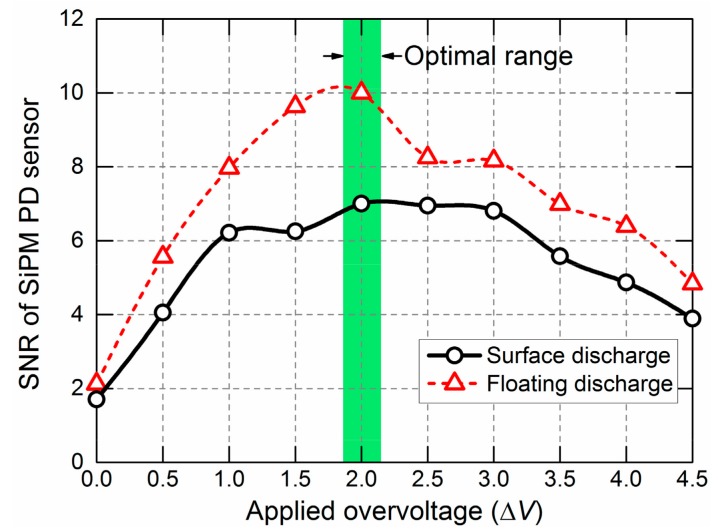
SNR of SiPM PD detection vs. applied over-voltage. The operating ambient temperature is approximately 25 °C. Surface discharge and floating discharge in an atmosphere of air are employed as PD light sources. The distance between the sensor and the PD source is 270 mm.

As described above, the over-voltage applied on the SiPM sensor has an essential impact on PD detection, and thus to make the SiPM-based PD detection work in room temperature conditions with an acceptable SNR, the applied over-voltage should be chosen appropriately, namely, the PDE (i.e., sensitivity) and DCR (i.e., device noise) of the SiPM sensor should be balanced at an appropriate applied voltage. For the practical PD detection, the applied over-voltage was determined experimentally. Two types of PDs (“floating discharges” and “surface discharges” (defined in Section 2.3)) in an atmosphere of air are used as the light sources and are maintained at stable PD magnitudes (for floating discharges, 740 pC, and for surface discharges, 520 pC) under a 50 Hz AC voltage (several kV). The operating ambient temperature is approximately 25 °C. The distance between the sensor and the PD source is 270 mm. The SNRs of PD detection are calculated by equation dividing the Weibull mean of the PD magnitude over a total 500 AC cycles to the maximum background noise level at the applied voltages from *U*_bias_+0 V to *U*_bias_+4.5 V. The variations of the SNR of the SiPM with the over-voltage are shown in Figure 1. Finally, the applied over-voltage was set at 2.0 V. For the PDs in other different gases and operating temperature, the applied voltages should be re-adjusted for the optimized SNRs.

**Figure 2:**
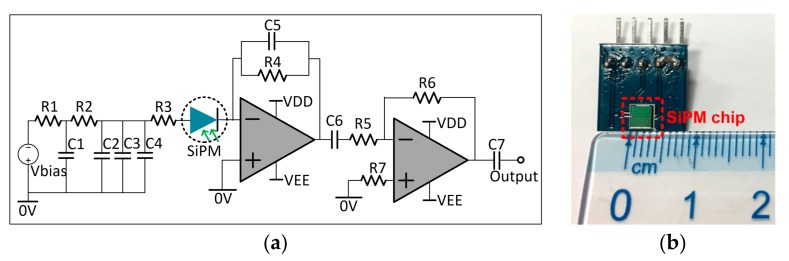
Silicon photomultiplier (SiPM)-based partial-discharge (PD) detector. (**a**) Simplified read-out circuit; (**b**) SiPM sensor used in the test. C1 = C2 = C3 = C4 = 10 nF; C5 = 100 pF; C6 = C7 = 0.1 μF; R1 = R2 = R5 = 50 Ω; R3 = R7 = 1 kΩ; R4 = 100 Ω; R6= 0.3 kΩ. The SiPM sensor in the circuit is the SensL-MicroFJ-30035-TSV model, which has 5676 microcells in a 3.07 × 3.07 mm^2^ active area, a broad response spectral range of 200–900 nm, a peak PDE of 51% at 420 nm and a bias point of voltage of 25 V.

To couple the photon-current induced by PD in a wide range with a high gain, a read-out circuit of the PD detector is designed with a transimpedance amplifier cascaded with a voltage amplifier, as shown in Figure 2a. A 3 × 3 mm^2^ SiPM sensor is used in this study, as shown in Figure 2b. Furthermore, the width of the PD pulses coupled by the detector (i.e., pulse resolution) is determined by the time constant of the circuit, which can be adjusted by changing the integral impedance in the transimpedance amplifier. 

**Figure 3:**
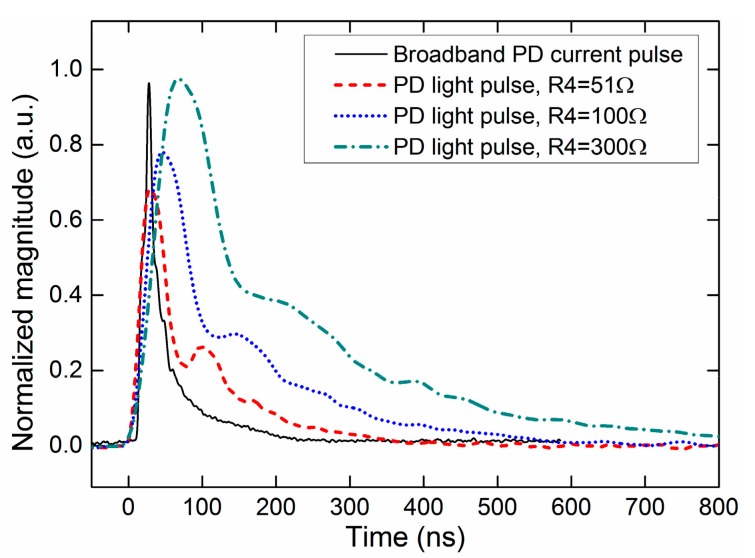
SiPM PD light pulse waveforms coupled with different external integral impedances in the primary stage. The PDs are excited by a positive polarity point in air.

The waveforms of the SiPM PD light pulses coupled with different integral impedances of the primary amplification stage are shown in Figure 3. The broadband current measurement indicates that the physical process of a PD event (positive point) lasts approximately 150 ns in air with a rise-time of 30 ns and a half-fall time of 60 ns. An increase in the integral impedance can increase the signal amplitude and reduce the required sampling rate, but decrease the pulse time resolution. In PD measurement of this study, R4 in the integral impedance is chosen as 100 Ω to obtain an acceptable pulse resolution, as well as to match the sampling module.

### 2.2. Synchronous PD Measurement Setup

**Figure 4:**
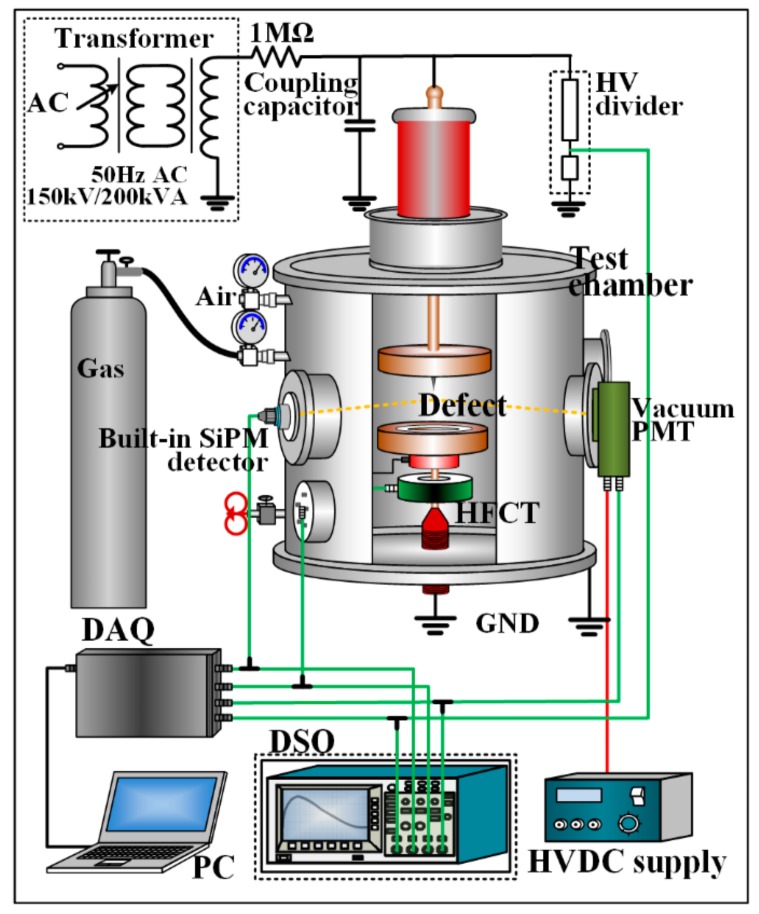
Synchronous PD measurement system.

To evaluate the SiPM PD detection performance, a HFCT (Pearson 6585; 400 Hz–250 MHz) and a stochastic PD recorder are used in the synchronous PD measurement, as shown in Figure 4. In addition, a vacuum PMT (Zolix. S1-CR131; response range of 180–920 nm; and, pulse response of 2.0 ns) is used for comparison with SiPM. All of the sensors are installed on a stainless-steel test chamber, which can withstand a voltage up to 150 kV (50-Hz AC peak). The built-in SiPM PD sensor is installed on the test chamber through an epoxy flange aiming to the PD source. The PMT is installed outside the synthetic fused silica glass window of the test chamber (above 85% transmittance from 175 nm onward and 90% from 220 nm onward). To couple the high-frequency PD current pulses, HFCT is installed around the grounding wire of the test object, and a 1500-pF coupling capacitor is connected in parallel with the test chamber. All of the current and light PD signals are collected by a multichannel PD recorder (developed based on a high-speed digital acquisition card (DAQ)) and a high-speed digital oscilloscope (DSO) (Lecroy; four channels; analog bandwidth of 600 MHz; sampling rate of 10 GS/s) simultaneously. A 150 kV/200 kVA no-corona 50 Hz AC transformer is used as the high-voltage (HV) power supply. 

### 2.3. Defect Models in Gas Insulation

PD activities caused by defects in the gas insulation can be approximately classified into three categories: point discharges in gases, discharges along the insulator surfaces, and gap sparks with floating conductors. According to their underlying mechanisms and detectabilities, the major discharge types are summarized in Table 1. The applied electric field strength is dominant in the transitions from one type of discharge to another. The weakest discharges are the emissions of charge carriers from the insulator surfaces and conductors [21], which always accompany electroluminescence phenomena. These types of discharges are characterized by notably low and quasi-continuous currents, which are undetectable by the common PD measuring techniques. With the increase in the applied electric field, another class of discharges with relatively low intensity, such as Townsend discharges [22] and swarming pulsive microdischarges (SPMD) [23], can be observed in highly sensitive laboratory experiments. The streamer, leader, and spark types of discharges are the most common types in gas insulation systems at relatively high-electric fields. They are relevant for the insulation status and necessarily detectable by PD detection.

**Figure 5:**
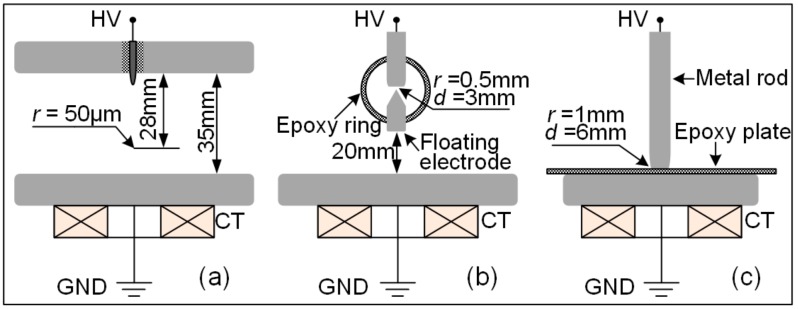
Configurations of the partial-discharge (PD) defect models. (**a**) Metal needle in a quasi-uniform background field; (**b**) Floating-potential metal particle; (**c**) Rod electrode on an epoxy insulator.

Considering the nature of two or three major dielectric boundaries (interfaces) that limit the discharges and the contribution of the electric field to the discharge excitation, three typical models were made for the test: a fixed metal protrusion on a conductor (Figure 5a), a floating metal particle near the electrode (Figure 5b) and a metal particle on an insulator surface (Figure 5c). For ease of description, the PDs excited by these three models are named “point discharge”, “floating discharge” and “surface discharge”, respectively. The artificial defect was placed in the high-voltage test chamber.

## 3. Results and Discussion

To investigate the actual performance of the SiPM-based PD detection, comparative studies were performed for the SiPM, conventional PMT, and wideband current pulse measurement in terms of sensitivity, pulse resolution, linear dependence of PD severity, and EM interference immunity. Furthermore, the stochastic features of the PD light intensities detected by the SiPM sensor were analyzed using the PRPD pattern.

### 3.1. Typical Output PD Pulses

**Figure 6:**
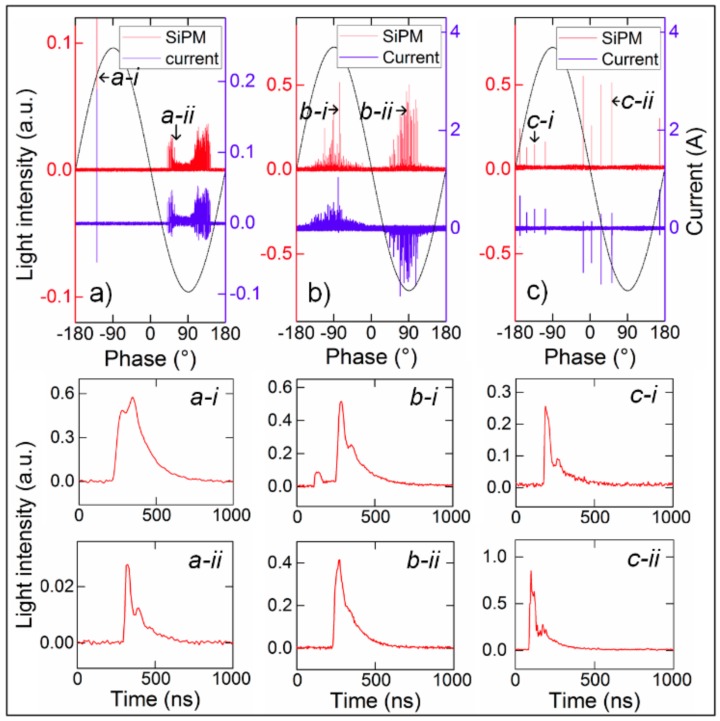
SiPM-detected light pulses with different types of PDs in air: (**a**) point discharges; (**b**) surface discharges; and, (**c**) floating discharges.

The partial discharge induced by a homogeneous field is a type of self-maintained intermittent discharge. All discharge-related processes including ionization, excitation, recombination, cathode photoionization, and secondary electron emission are accompanied by photon emission and absorption. During discharge events, certain proportions of photons in the above active processes escape from the discharge active region and can be detected by the photoelectric devices. The output light pulses of SiPM show different pulse waveforms and pulse sequences for different types of defect models. Figure 6 shows the light pulses and current pulses, which were synchronously detected in three cases of PD sources (point discharges, surface discharges, and floating discharges). Because of different distributions of the electric field and dielectric interfaces involved in the discharges, PDs vary in current pulses and accompanying light emission pulses in terms of PD magnitudes (current magnitude in unit of A, light intensity in arbitrary unit (a.u.)), PD sequences and pulse waveforms, as shown in Figure 6 and their subfigures. With the advantages of a high sensitivity and high response rate, SiPM sensor can reflect the variation of light during a PD process with high time/magnitude resolution. The more intrinsic features of the PDs in different modes can be obtained by SiPM based PD detection even in practical application, which can hardly be achieved by frequency bandwidth/sensitivity-limited current detections. In general, the comparison of the current pulses and light pulses indicates that the SiPM PD detection has an identical capability to the current pulse detection in characterizing PD activities.

### 3.2. Basic Performances of the SiPM-Based PD Detection 

#### 3.2.1. Lower Limit of Detection

**Figure 7:**
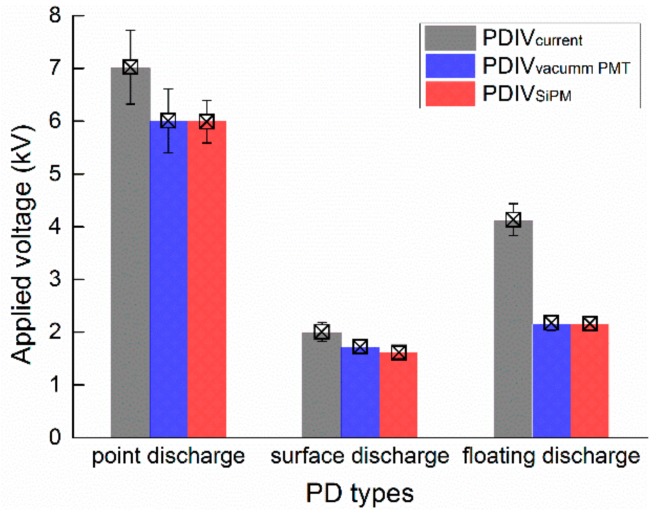
Partial-discharge inception voltages (PDIVs) of different defect models identified by SiPM, photomultiplier tube (PMT), and current pulse detections. The applied over-voltages on SiPM and PMT are 2.5 V and 800 V, respectively. The noise level of the current pulse detection in the laboratory condition is below 2 pC. The operating ambient temperature is approximately 25 °C.

When considering the PD diagnosis, PDs are expected to be detected in the initial phases and mainly to prevent a total breakdown of the insulation system. Therefore, the lower limit of PD detection is crucial for the insulation examination. In this study, partial-discharge inception voltages (PDIVs) of different PD models, as described in Section 2.3, are identified by SiPM, PMT, and current PD detections, as shown in Figure 7. The optical detections can respond to PDs at relatively lower applied voltages than the current pulse detection for all of the cases of PD sources, particularly the floating potential discharges. Because of the high quantum efficiency in a wide response spectral range, the SiPM-based PD detection has an almost identical capability to vacuum PMT in responding to the notably weak PDs in air and some electronegative gases with no additional signal amplification.

#### 3.2.2. Pulse Resolution Time

**Figure 8:**
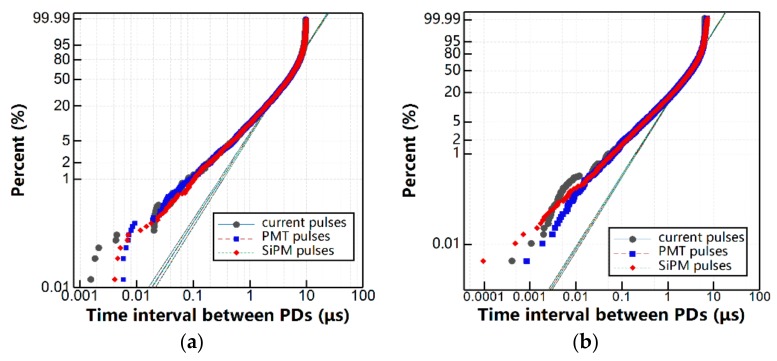

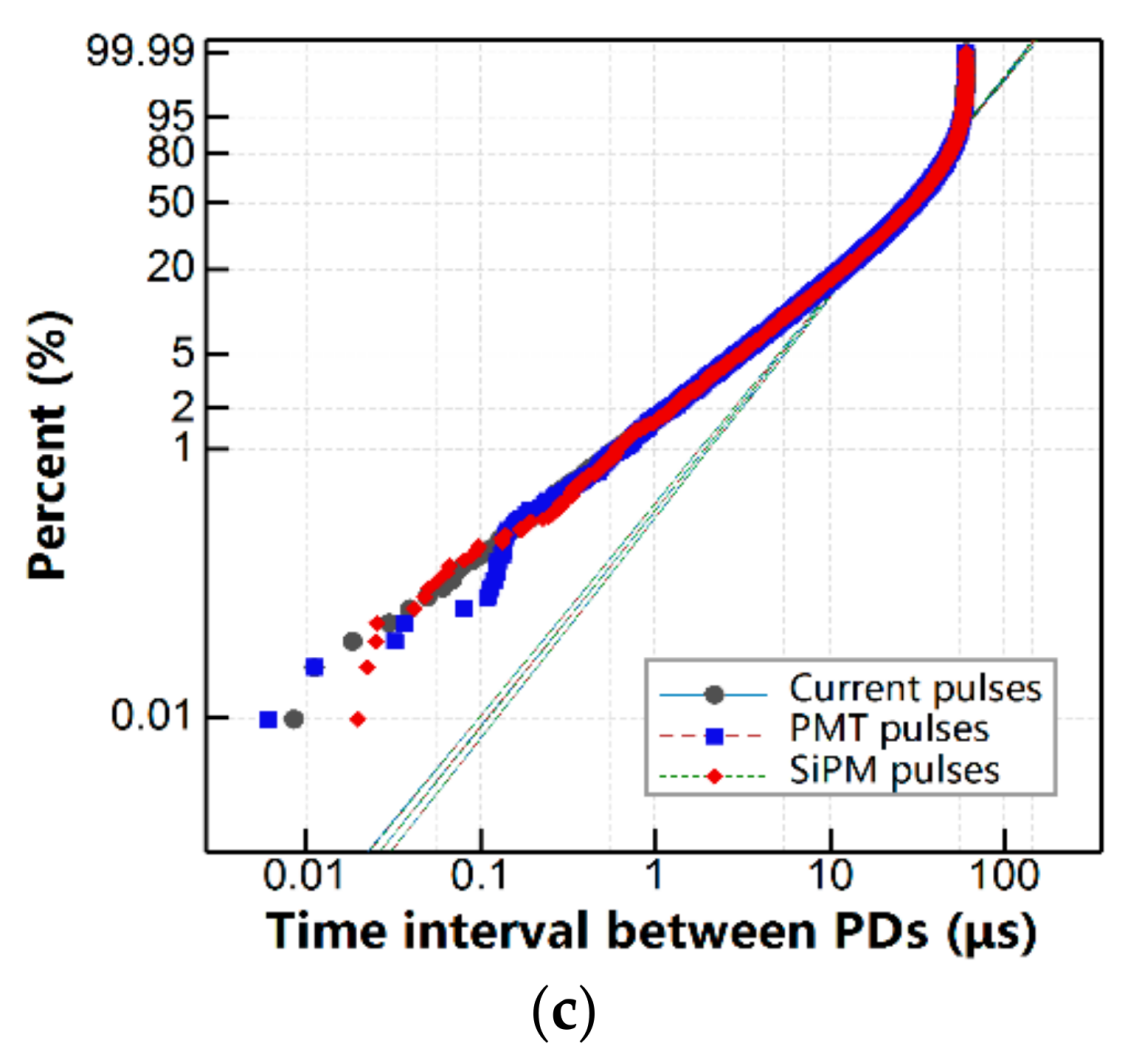
Weibull distributions of the time intervals between successive PD events recorded by high-frequency current transformer (HFCT), PMT, and SiPM. (**a**) Negative point discharge; (**b**) Surface discharge; and, (**c**) Floating discharge.

A good pulse resolution of a PD detection system ensures that most PD events are precisely recorded, particularly for stochastic PD analyses. The pulse time resolution of the SiPM PD detection actually depends on the response time for the microcell to work at Geiger avalanche mode [13], recovery time to recharge, and external matching circuit. To compare the SiPM PD detection with the conventional current pulse response and PMT detection in terms of time resolution, the time intervals between successive PD events, which are recorded by complete stochastic measurements, are analyzed by Weibull probability distributions, which are calculated by Equation (4) [26], as shown in Figure 8.

(4) $$\ln(-\ln(1-F(\Delta t)))=\beta \ln\Delta t-\beta \ln\Delta t_{0},$$

Here, F is the probability as a function of the scale parameters, Δ*t*, and shape parameter, *β*. 

It indicates that for all the cases of defect models investigated, the Weibull distribution of the time interval recorded by SiPM-based PD detection almost overlaps those recorded by HFCT and vacuum PMT, except in the time range below 0.1 μs. Hence, the SiPM-based PD detection can accurately record the overwhelming majority of PD events for all of the types of PDs. Weibull parameters of the time intervals of PD pulses detected by HFCT, PMT, and SiPM, including scale parameter (i.e., Weibull time interval), Δ*t*, shape parameter, *β*, sample number, and *N* and error value (ER)) are listed in Table 2. The comparison indicates that the pulse intervals recorded by SiPM-based PD detection, have strongly consistent Weibull parameters (e.g., shape (*β*) and scale (*α*)) with those of HFCT and PMT; thus, SiPM can also be applied to stochastic PD detection with a high accuracy in PD event recognition.

#### 3.2.3. EM Interference Immunity in PD Detection

**Figure 9:**
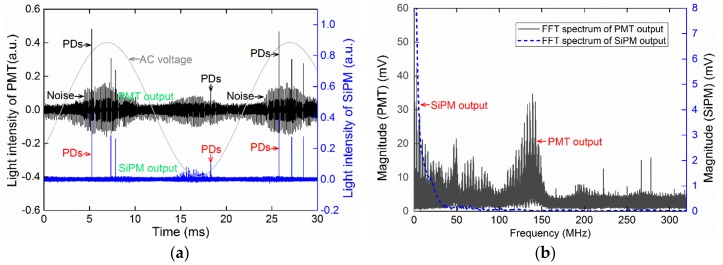
PD outputs of PMT and SiPM sensors in identical electromagnetic (EM) noise conditions. (**a**) Signals of SiPM and PMT in time domain; (**b**) FFT spectra of SiPM and PMT outputs. A high-frequency electromagnetic switch in operation is used as the actual noise source and placed near the PMT and SiPM sensors with a distance of approximately 1.0 meter.

The sensitivity of the SiPM PD detection is high because of the good interference immunity of the SiPM sensor. Unlike the vacuum PMT, the solid-state APD units in SiPM sensors are insensitive to magnetic fields in all directions, which impart a low level of device noise in the actual PD detection, particularly in strong EM environments. In comparison, the SiPM outperforms the PMT in terms of EM interference immunity. Figure 9 shows an example of a comparison between PD signals simultaneously obtained by PMT and SiPM in identical strong noise conditions. A running high-frequency electromagnetic switch is used as the noise source and placed near the optical sensors with a distance of approximately 1.0 m. For the practical applications of the PD detection methods, such as UHF, HFCT, and AE methods, the signal processing approaches such as hardware filters, digital filters, wavelet denoising and clustering denoising techniques, are necessary in noise suppression and PD recognition. It is demonstrated that the SiPM-based PD detection has a significant EM interference immunity even without any above mentioned signal processing, which contributes to a relatively higher SNR in the actual application. The good EM interference immunity of the SiPM-based detection is benefit from the insensitivity to magnetic field of silicon-based solid state sensor. When comparing with SiPM, PMT is easily vulnerable to EM interference because the photoelectron multiplication process of PMT is sensitive to the electric and magnetic field bias.

### 3.3. Quantitative Relationship between the SiPM Output and the Current Pulse

**Figure 10:**
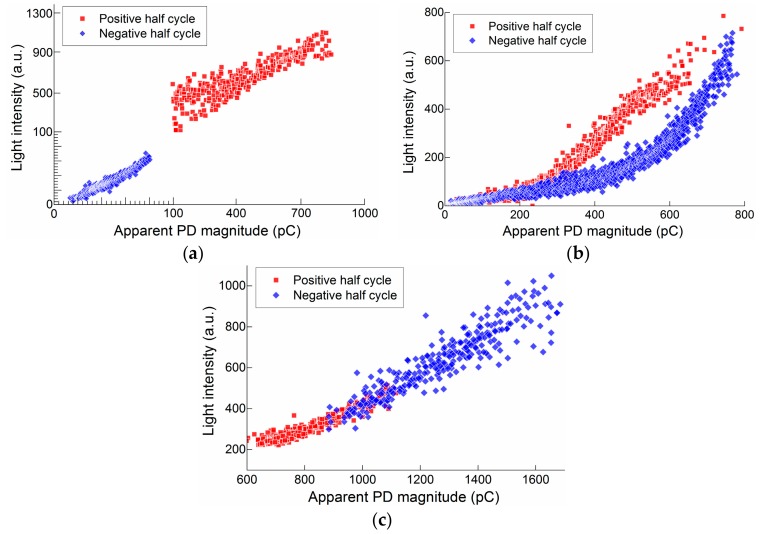
Light intensity detected by SiPM vs. apparent PD magnitude. (**a**) Point discharge; (**b**) Surface discharge; and, (**c**) Floating discharge. The tests are performed in air condition. Over-voltages applied on SiPM and PMT are 2.0 V and 600 V, respectively.

A monotonic relationship between the signal response and the strength of a physical object is the premise for building a measurement system. The intensity of the light emitted from the discharge is positively associated with the quantity of PD charge and depends on the ratios among the probabilistic cross sections of the excitation, ionization, recombination, and attachment during a complete discharge process [27]. In general, the radiation spectrum and light intensity are closely related to the specific electric field distribution and the involved interfaces of the dielectrics. Figure 10 shows the relationships between the light intensity detected by SiPM and the apparent PD magnitude that was detected by a HFCT for all of the investigated types of PDs. For different types of insulation defect and for the same defect, but under different polarities of applied voltage, the PDs have the different active ranges in PD charge as well as in light intensity. For point discharge, although the PD active range under negative half cycle of the applied voltage (tens of pC) is significantly different from that under the positive half (hundreds of 100 pC), the light pulse magnitude is linearly proportional to the increase of apparent PD magnitude for both negative and positive half cycles, as shown in Figure 10a. For surface discharge, PDs are active in notably wide ranges from several pC to hundreds of pC for both negative and positive half cycles. The relationships between light intensity and PD charge at positive and negative half cycles remain consistent in a relatively low active range, but present different trends as the PDs are more active, as shown in Figure 10b. This result is considered as related to the variations of ratios among the probability cross sections of discharges involved in photon emission processes, which are caused by the transitions of mechanisms underlying PD activities. When compared to the former types of PDs, the floating discharge is more active once the applied voltage exceeds the PDIV, particularly in the negative half cycles, which causes the very intensive light emission. Statistically, the light pulse magnitudes in both half cycles linearly increase with the increase in apparent PD magnitude in the entire PD active range, as shown in Figure 10c. In general, the magnitude of the SiPM output is positively correlated with the apparent PD magnitude, which indicates that the SiPM-based PD detection can accurately characterize the PD severity.

### 3.4. Stochastic PD Detection

**Figure 11:**
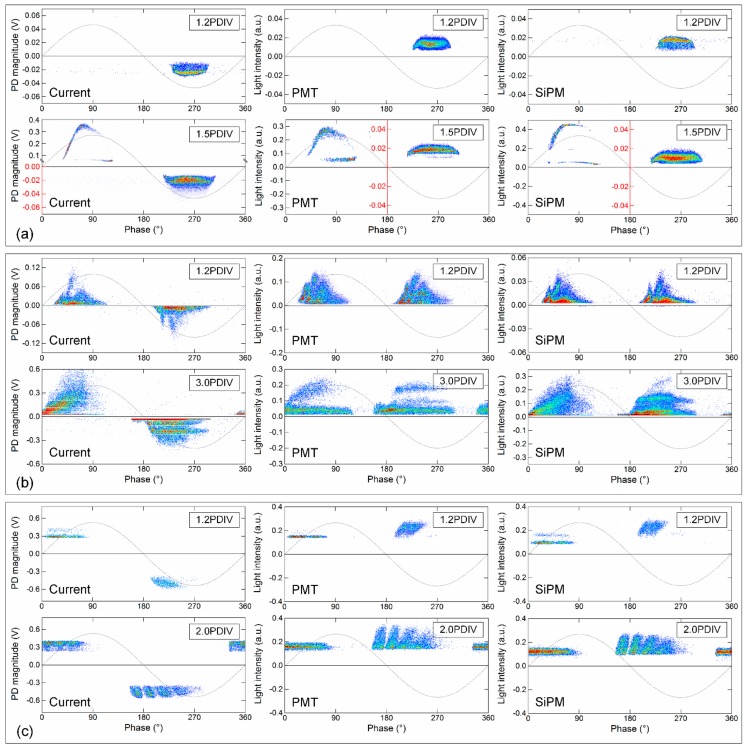
Phase-resolved PD pattern (PRPD) patterns of PD light intensities detected by HFCT, PMT and SiPM at different levels of PD activity. (**a**) Point discharge; (**b**) surface discharge; (**c**) floating discharge. The tests are performed in air condition. The over-voltages applied to SiPM and PMT are 2.5 V and 800 V, respectively. The noise level of the PD current pulse detection is below 0.5 pC.

When an alternating current (AC) electric field is applied, the discharge activities periodically vary in terms of time lag and magnitude, based on which the PRPD analysis was proposed for PD diagnosis and defect recognition in insulation systems under AC HV [28]. The PD data in a certain number of applied voltage cycles are plotted on the phase axis in one voltage cycle. This phase-related depiction of PDs provides additional information, including the physical properties and stochastic behaviors of the discharges. Similarly, the PRPD pattern can be used to analyze stochastic light pulses. In this case, the applied voltage peak, *U*, corresponding phase degree, *ϕ*, and light pulse magnitude, *I*, are simultaneously recorded for thousands of AC cycles with optical detection to plot the PRPD patterns. Figure 11 shows the obtained PRPDs for different types of defects at the PD initial phases and PD intensive phases. By comparison, the PRPD results of the SiPM and current pulse measurement are considerably consistent with each other in terms of both profile and distribution intensity; the consistency is even higher than that between the PMT and the current from the HFCT. This good performance of SiPM-based PD detection results from the high pulse time resolution and high linearity with the PD magnitude of SiPM, as mentioned in Section 3.2 and Section 3.3. It indicates that the main distribution ranges of the SiPM PRPDs are consistent with those of current PRPDs, especially for the defect models of surface discharge and floating discharge. Consequently, the conventional PD diagnosis and defect recognition approaches can also be applied to SiPM-based PD monitoring.

## 4. Conclusions

In this paper, the SiPM sensor is introduced for PD detection. The basic performances of SiPM in PD detection were investigated by comparisons with the current pulse detection and conventional PMT detection. Based on the experimental studies, the main conclusions are as follows.

The PDE (i.e., sensitivity) and DCR (i.e., device noise) of the SiPM sensor should be balanced by selecting an appropriate applied over-voltage to achieve an acceptable sensitivity and a relatively low noise level in room temperature conditions. The increase in coupling impedance can increase the sensitivity and reduce the required sampling rate, but the pulse time resolution is also decreased.

Weibull parameters of the pulse intervals recorded by SiPM-based PD detection, including the shape (*β*) and scale (*α*), are highly consistent with those of HFCT and PMT, which indicates that SiPM can be applied to stochastic PD detection with a high-accuracy in PD event recognition.

Because the high quantum efficiency covers a wide response spectral range, the SiPM-based PD detection almost has the identical capability to vacuum PMT in responding to notably weak PDs with no additional signal amplification.

In general, the magnitude of the SiPM output is positively correlated with the apparent PD magnitude. In the PD active ranges of PDs, the relationship between the light relative intensity and the apparent PD magnitude can be roughly described by a single or piecewise approximate linear form, which implies that the SiPM-based PD detection can accurately characterize the PD severity.

The SiPM-based and HFCT-based detections have considerably consistent PRPDs in terms of both profile and distribution intensity; this consistency is even higher than that between the PMT-based and HFCT-based detections. This good performance results from the high pulse time resolution and high linearity with the PD magnitude of SiPM.

Because of the additional advantages of SiPM-based PD detection, such as the small size, low power supply, and low cost, the built-in optical PD monitoring for gas-insulated system can be put into practical use in the near future.

## Figures and Tables

**Table 1:** Gaseous discharge types caused by insulation defects, which are summarized according to their intensities, features and detectabilities.

Discharge Type	Intensity	Feature	Detectability	
			Current	Light
Field emission	10^−10^, …10^−5^ A/m^2^ [24]	continuous	no	no
glow	~10 A/m^2^ [25]	continuous	no	yes
Townsend, SPMD	~100 μA [25]	pulsed, ~100 ns	yes	yes
streamer	~mA, >10 pC	pulsed, ~100 ns	yes	yes
leader	~0.1, …~10 A	pulsed, 0.01…1 ms	yes	yes
spark	~0.1, …~10 A	pulsed, 0.001…1 ms	yes	yes
partial arc	~1, …~10 A	pulsed to continuous	yes	yes

**Table 2:** Comparisons of Weibull parameters between the PD pulses detected by the current, PMT and SiPM.

Statistical Parameters (Sensor)	Δ*t*_0_	*β*	*N*	ER
Point discharge (HFCT)	5.367	1.626	4999	/
Point discharge (PMT)	5.354	1.630	4999	0.24%
Point discharge (SiPM)	5.358	1.631	4999	0.17%
Surface discharge (HFCT)	3.520	1.612	16,000	/
Surface discharge (PMT)	3.529	1.612	16,000	0.26%
Surface discharge (SiPM)	3.527	1.606	16,000	0.20%
Floating discharge (Current)	33.24	1.610	7500	/
Floating discharge (PMT)	33.24	1.610	7500	0.00%
Floating discharge (SiPM)	33.24	1.613	7500	0.00%
